# The Study of a 231 French Patient Cohort Significantly Extends the Mutational Spectrum of the Two Major Usher Genes *MYO7A* and *USH2A*

**DOI:** 10.3390/ijms222413294

**Published:** 2021-12-10

**Authors:** Luke Mansard, David Baux, Christel Vaché, Catherine Blanchet, Isabelle Meunier, Marjolaine Willems, Valérie Faugère, Corinne Baudoin, Melody Moclyn, Julie Bianchi, Helene Dollfus, Brigitte Gilbert-Dussardier, Delphine Dupin-Deguine, Dominique Bonneau, Isabelle Drumare, Sylvie Odent, Xavier Zanlonghi, Mireille Claustres, Michel Koenig, Vasiliki Kalatzis, Anne-Françoise Roux

**Affiliations:** 1 Molecular Genetics Laboratory, University of Montpellier, CHU Montpellier, F-34000 Montpellier, France; l-mansard@chu-montpellier.fr (L.M.); d-baux@chu-montpellier.fr (D.B.); christel.vache@inserm.fr (C.V.); valerie.faugere@inserm.fr (V.F.); corinne.baudoin@inserm.fr (C.B.); melody.moclyn@chu-lyon.fr (M.M.); j-bianchi@chu-montpellier.fr (J.B.); mireille.claustres@inserm.fr (M.C.); michel.koenig@inserm.fr (M.K.); 2 Institute for Neurosciences of Montpellier (INM), University of Montpellier, Inserm, F-34000 Montpellier, France; i-meunier@chu-montpellier.fr (I.M.); m-willems@chu-montpellier.fr (M.W.); vasiliki.kalatzis@inserm.fr (V.K.); 3 National Reference Centre for Inherited Sensory Diseases, University Montpellier, CHU Montpellier, F-34000 Montpellier, France; c-blanchet@chu-montpellier.fr; 4 Oto Laryngology Department, University of Montpellier, CHU Montpellier, F-34000 Montpellier, France; 5 Medical Genetics Department, University of Montpellier, CHU Montpellier, F-34000 Montpellier, France; 6 Reference Center for Rare Affections in Ophthalmology Genetics (CARGO), Institute of Medical Genetics of Alsace, University of Strasbourg, CHU Strasbourg, F-67000 Strasbourg, France; helene.dollfus@chru-strasbourg.fr; 7 Medical Genetics Department, University of Poitiers, CHU Poitiers, F-86000 Poitiers, France; brigitte.gilbert-dussardier@chu-poitiers.fr; 8 Medical Genetics Department, University of Toulouse, CHU Purpan, F-31000 Toulouse, France; dupin-deguine.d@chu-toulouse.fr; 9 Medical Genetics Department, University of Angers, CHU Angers, F-49000 Angers, France; dobonneau@chu-angers.fr; 10 Vision and Neuro-Ophthalmology Department, University of Lille, CHU Lille, F-59000 Lille, France; Isabelle.DRUMARE@CHRU-LILLE.FR; 11 Clinical Genetics Service, University Hospital, Genetics and Development Institute of Rennes IDGDR, UMR6290 University of Rennes, F-35000 Rennes, France; sylvie.odent@chu-rennes.fr; 12 Center of Competence for Rare Diseases, Jules Verne Clinic, F-44000 Nantes, France; dr.zanlonghi@gmail.com

**Keywords:** Usher syndrome, retinitis pigmentosa, hearing loss, *MYO7A*, *USH2A*, pathogenic genotype, ACMG classification, deep intronic variant

## Abstract

Usher syndrome is an autosomal recessive disorder characterized by congenital hearing loss combined with retinitis pigmentosa, and in some cases, vestibular areflexia. Three clinical subtypes are distinguished, and *MYO7A* and *USH2A* represent the two major causal genes involved in Usher type I, the most severe form, and type II, the most frequent form, respectively. Massively parallel sequencing was performed on a cohort of patients in the context of a molecular diagnosis to confirm clinical suspicion of Usher syndrome. We report here 231 pathogenic *MYO7A* and *USH2A* genotypes identified in 73 Usher type I and 158 Usher type II patients. Furthermore, we present the ACMG classification of the variants, which comprise all types. Among them, 68 have not been previously reported in the literature, including 12 missense and 16 splice variants. We also report a new deep intronic variant in *USH2A*. Despite the important number of molecular studies published on these two genes, we show that during the course of routine genetic diagnosis, undescribed variants continue to be identified at a high rate. This is particularly pertinent in the current era, where therapeutic strategies based on DNA or RNA technologies are being developed.

## 1. Introduction

Usher syndrome refers to recessively inherited disorders associating hearing loss (HL), retinitis pigmentosa (RP) and, sometimes, vestibular areflexia. It represents the most frequent form of blindness–deafness, with a prevalence of 1/25–30,000 worldwide [1]. Three clinical subtypes (USH1-3) are distinguished based on the severity and progression of the HL and the presence or absence of vestibular areflexia. Usher syndrome type I (USH1) is the most disabling form, with congenital profound HL and vestibular dysfunction in most cases. RP will develop progressively from the first decade of life.

**Figure A1:**
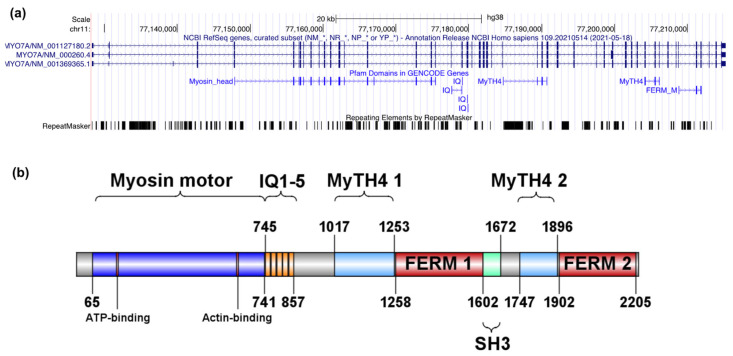
Genomic and encoded protein structure of *MYO7A*. (**a**) Genomic representation of the *MYO7A* gene and the associated Pfam (the protein families database; https://pfam.xfam.org) domains as indicated by UCSC Genome Browser (https://tinyurl.com/fbjwskcz, accessed on 8 December 2021). (**b**) Schematic representation of the UNIPROT myosin VIIa domains (Q13402, 2215 residues) and their localization within the protein.

**Figure A2:**
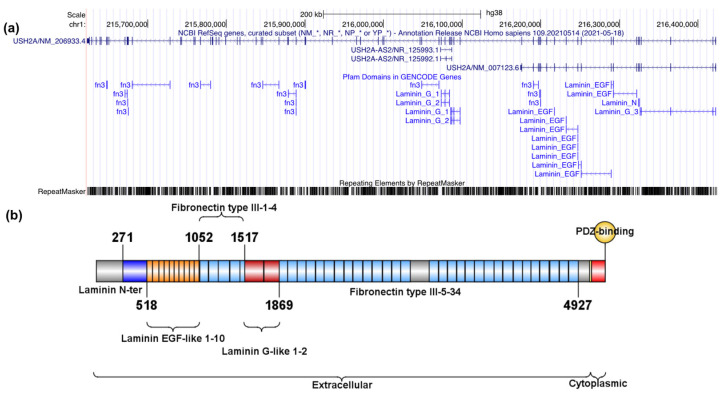
Genomic and encoded protein structure of *USH2A*. (**a**) UCSC Genomic representation of the *USH2A* gene and the associated Pfam (the protein families database; https://pfam.xfam.org) domains as indicated by UCSC Genome Browser (https://tinyurl.com/2p9aexr5 accessed on 8 December 2021). (**b**) Schematic representation of the UNIPROT usherin domains (O75445, 5202 residues) and their distribution along the protein.

Among the five causative genes known to date, *MYO7A* (OMIM 276903, Figure A1) represents the most prevalent gene, causing up to 60–70% of USH1 cases [2,3,4]. Usher syndrome type II (USH2) is the most frequent form with congenital moderate-to-severe HL [5]. RP can develop from the second decade onwards. As for USH1, restriction of the visual field and night blindness are the first symptoms. Mutations in three known genes cause USH2, and *USH2A* (OMIM 608400, Figure A2) represents the most prevalent gene accounting for up to 90% of the cases [4,6,7]. Of note, *MYO7A* is also involved in nonsyndromic (NS) HL, with an autosomal dominant or recessive pattern of inheritance [8,9]. Similarly, *USH2A* is implicated in isolated autosomal recessive RP and represents up to 30% of the cases [10].

Numerous pathogenic variants have been identified in *MYO7A* and *USH2A* (384 and 1146, respectively, recorded in the Global Variome LOVD Shared instance, as of October 2021). These variants are distributed throughout the 49 *MYO7A*- and 72 *USH2A*-coding exons. In this respect, and because of the overall genetic heterogeneity of Usher syndrome, molecular diagnosis has tremendously improved in recent years thanks to the development of Massively Parallel Sequencing (MPS) in diagnostic laboratories [11,12]. This approach has highlighted the existence of rare atypical Usher cases, as some patients presenting with the USH2 phenotype can harbor variants in USH1 genes [4], and conversely, patients with the USH1 clinical phenotype can have mutations in *USH2A* [11].

In this paper, we present 231 new pathogenic genotypes in the two major USH genes, 73 in *MYO7A* and 158 in *USH2A*. Among the 462 identified alleles, 48% are unique and 15.2% are novel. Of all the types of alterations identified, the two genes are particularly prone to missense alterations, as they represent 38.4% and 26.4% of the causative alleles for *MYO7A* and *USH2A*, respectively. Consequently, additional prediction studies are needed to ascertain their deleterious effect, highlighting the ongoing challenge of diagnosis to provide proper genetic counselling. 

## 2. Results

### 2.1. MYO7A and USH2A Variant Identification and Spectrum

#### 2.1.1. Description of *MYO7A* and *USH2A* Alterations

**Figure 1:**
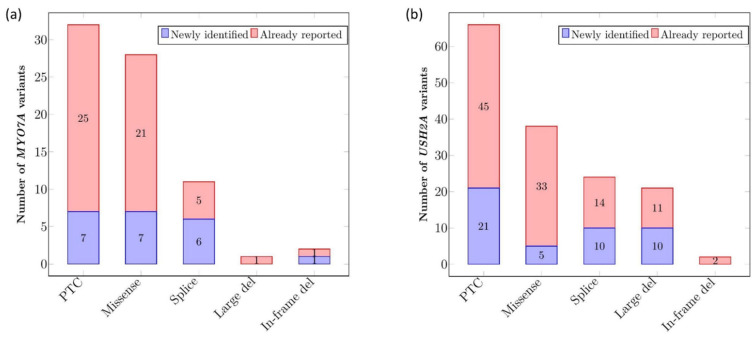
Types of variants in *MYO7A* (**a**) and *USH2A* (**b**) and proportion of novel alterations per type. The number of newly identified (purple bars) or already reported (pink bars) variants per alteration type are indicated within the bars. A total of 74 variants were identified for *MYO7A* (**a**) and 151 variants for *USH2A* (**b**). All variants were counted once, independently of their frequency in the cohort.

*MYO7A* and *USH2A* alterations are listed in Tables S1 and S2. In total, 74 different variants were identified in *MYO7A* (Figure 1a). Among them, 43.2% (n = 32) were predicted to give rise to Premature Termination Codons (PTCs) and 37.8% (n = 28) were missense variations. Splicing alterations represented 14.9% (n = 11), in-frame deletions (under 30 bp in this cohort) 2.7% (n = 2) and large deletions (defined here as larger than 100 bp) 1.4% (n = 1) of the identified variants. Considering *USH2A,* a total of 151 different variants were identified (Figure 1b). Variants predicted to result in PTCs were the most frequently encountered type (43.7%, n = 66), followed by missense variations (25.2%, n = 38), splicing alterations (15.9%, n = 24), large deletions (13.9%, n = 21) and in-frame deletions 1.3% (n = 2).

The classification of variants was performed following ACMG/AMP recommendations ([13], Table A1). In most cases, it led to the definition of class 4 or class 5 variants (defined as likely pathogenic and pathogenic, respectively, see Tables S1 and S2). However, we report several class 3 variants, defining variants of unknown clinical significance. Detailed ACMG criteria of classification for such variants can be found in the MobiDetails website (see methods). All these class 3 variants have been included in the current report because, while considered as of unknown significance following the current ACMG/AMP guidelines, the majority of gathered evidence was in favor of pathogenicity. As an example, the newly described *MYO7A* c.1517T>A-p.(Ile506Asn) variant is located within the motor domain of the protein (Figure A1). Conservation among vertebrates as visualized on UCSC shows only aliphatic hydrophobic residues at this position (5 Val, 1 Leu and 94 Ile). Consequently, most missense predictors consider this variant as deleterious. The wild-type Ile residue lies within an alpha helix, and participates in a hydrophobic pocket, as visualized on https://alphafold.ebi.ac.uk/entry/Q13402 (accessed on 8 December 2021), by selecting the appropriate residue. Introducing a polar Asn residue is highly likely to destabilize the pocket and to have an impact at least on the local structure. Application of the corresponding ACMG criteria PM1 (located in a mutational hot spot and/or critical and well-established functional domain), PM2 (absent from controls (or at extremely low frequency if recessive) in population studies) and PP3 (multiple lines of computational evidence support a deleterious effect) leads to the classification of this variant as class 3, despite evidence of pathogenicity. A familial segregation study was not feasible for this patient, who also carries a PTC in *MYO7A*.

Finally, we report 21 novel *MYO7A* alleles including 7 PTCs, 7 missense variants, 6 splicing variations and one in-frame deletion. None of them have been found more than once. A total of 47 novel *USH2A* alleles are described, including 21 PTCs, 5 missense, 10 large deletions and 10 splicing alterations. The proportion of these novel alleles compared to the total alleles is presented in Figure 1. ACMG classification was applied to all of them (Tables S1 and S2).

#### 2.1.2. Mutational Spectrum of the Cohort

**Figure 2:**
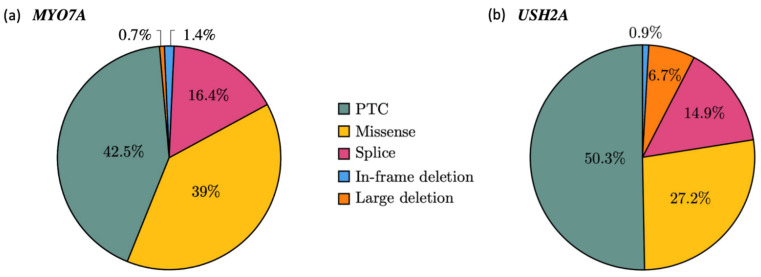
Mutational spectrum in *MYO7A* (**a**) and *USH2A* (**b**). Pie charts showing the allelic frequencies for each alteration type in the cohort; 146 alleles for *MYO7A* (**a**) and 316 alleles for *USH2A* (**b**) The variants were counted each time they occurred in the cohort.

Among the 146 *MYO7A* pathogenic alleles (73 patients), PTCs represented 42.5% of the mutated alleles (n = 62), missense variations 39% (n = 57) and splicing alterations 16.4% (n = 24). Deletions were rare, with 1.4% (n = 2) of small in-frame events and one large deletion (0.7%) (Figure 2a). Similarly, the prevalence of each type of variation in *USH2A* revealed a majority of PTCs with 159 alleles of a total of 316 (50.3%), followed by missense alterations reported for 86 alleles (27.2%) and splicing alterations in 47 alleles (14.9%). Overall, 21 alleles consisted of large deletions (6.6%) and 3 others carried an in-frame deletion (0.9%) (Figure 2b). 

#### 2.1.3. Recurrence of Alterations

Several recurrent alterations or founder effects were identified for both genes. We did not consider the alleles present in the homozygous state in one patient of known or suspected consanguineous lineage as recurrent. For *MYO7A*, 22 recurrent variations were identified in at least two independent *MYO7A* alleles. The two most frequent were the well-known missense alterations, c.3719G>A - p.(Arg1240Gln) and c.6557T>C - p.(Leu2186Pro), which were detected in nine and eight alleles, respectively. 

Concerning *USH2A*, 37 different alterations were found to be recurrent, with an overwhelming majority carrying the c.2299delG - p.(Glu767SerfsTer21) variant found in 47 alleles, representing 14.2% of all the identified alterations. The second most frequent alteration was the deep intronic variant c.7595-2144A>G, known to introduce an out-of-frame pseudoexon between exons 40 and 41 [14], with 16 occurrences in this cohort (5.1%). The c.14803C>T - p.(Arg4935Ter) alteration was identified in nine alleles (2.9%) and completed the top three most frequent *USH2A* alleles.

#### 2.1.4. Identification of a Novel Deep Intronic Variation

**Figure 3:**
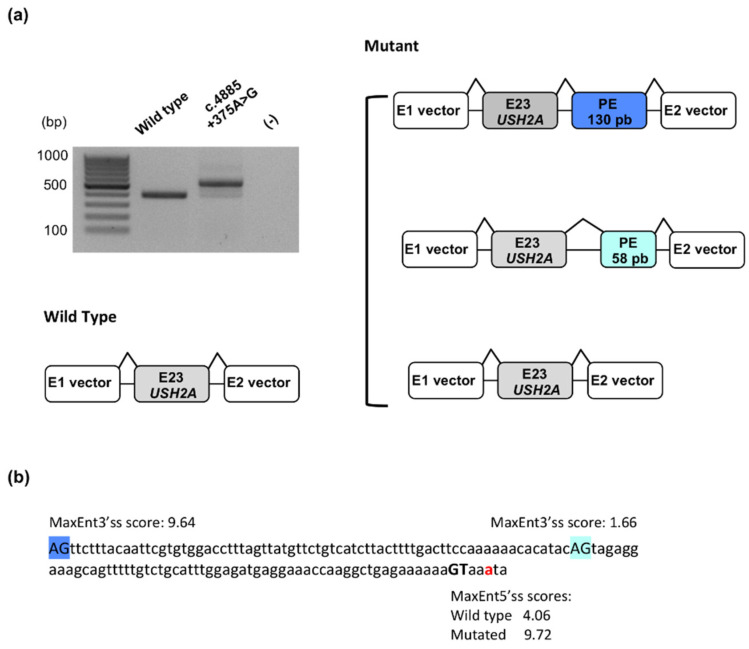
Minigene assay of the c.4885+375A>G *USH2A* variant. (**a**) Electrophoretic visualization of RT-PCR products amplified from wild-type and mutant constructs and schematic representation of the corresponding splicing patterns. White boxes represent the pSPL3 exons, grey boxes correspond to exon 23 of *USH2A* and dark or light blue boxes represent two pseudoexons (PEs) of 130 and 58 nucleotides, respectively. (**b**) Intronic sequence involved in the inclusion of the two observed pseudoexons. The c.4885 adenine is indicated with red font; the activated donor splice site is shown with bold black font and the two used acceptor splice sites are highlighted in dark or light blue. MaxEnt donor splice site strength scores (5′ss scores) and MaxEnt acceptor splice site strength scores (3′ss scores) are indicated for the three splice sites.

Because gene-panel screening performed on patient U1432 identified a single *USH2A* causal splice alteration c.949C>A - p.(=, Tyr318CysfsTer17) in the heterozygous state, sequencing of the whole *USH2A* gene was performed. We identified, in intron 23, the c.4885+375A>G substitution as a potential splice-altering variant via the activation of a cryptic 5′ donor splice site. This site would result in the inclusion of a pseudoexon in the synthetized transcripts. To confirm this predicted splicing defect, a minigene assay was conducted for the variant (Figure 3).

Hela cells transfected with the mutant minigene yielded a predominant aberrant transcript (Figure 3a). This one was larger than the RT-PCR product obtained from cells transfected with the wild-type construct, due to the inclusion of an out-of-frame pseudoexon of 130 nucleotides. Two distinct minor populations of transcripts were also detected, corresponding to correctly spliced transcripts and aberrant transcripts harboring an out-of-frame pseudoexon of 58 nucleotides. The two inserted pseudoexons (Figure 3b) used the same activated donor splice site (mutant MaxEnt score: 9.72; wild-type MaxEnt score: 4.06), but different cryptic acceptor splice sites (MaxEnt scores of 9.64 and 1.66 for the major and minor transcripts, respectively).

### 2.2. Spectrum of MYO7A and USH2A Pathogenic Genotypes

In order to unambiguously determine the genotypes, familial segregation was performed whenever possible. This included analysis of one or both parents. Overall, familial segregation was possible for 52% of the families, 52 mutated in *MYO7A* and 68 mutated in *USH2A*. We subsequently included solo patients as well, because the molecular findings were sufficiently convincing, while bearing in mind that for any further studies (genetic counseling and/or inclusion in clinical trials) segregation analyses would still be required.

#### 2.2.1. Pathogenic Classification of *MYO7A* and *USH2A* Genotypes 

Most of the reported genotypes involved a combination of two class 5 variants (39 in *MYO7A*, 89 in *USH2A*), two class 4 variants (9 in *MYO7A*, 15 in *USH2A*) or a combination of class 4/class 5 variants (22 in *MYO7A*, 42 in *USH2A*). However, in one *MYO7A* patient, genotype included a combination of class 3/class 4 (U1076) and in another three patients, a combination of class 3/class 5 (U1489, U1770, U2817). A total of 12 patients mutated in *USH2A* presented combinations involving one class 3. This includes three class 3/class 4 combinations, (U1359, U1370, U1888), eight class 3/class 5 (U1336, U1380, U1468, U1517, U1702, U1932, U1950, U2151) and one class 3/class 3 (U1530). Expectedly, all reported class 3 variants in either of the two genes were missense variants or small in-frame deletions, and fifteen of them were identified in solo patients.

#### 2.2.2. Genotype Spectrum 

**Figure 4:**
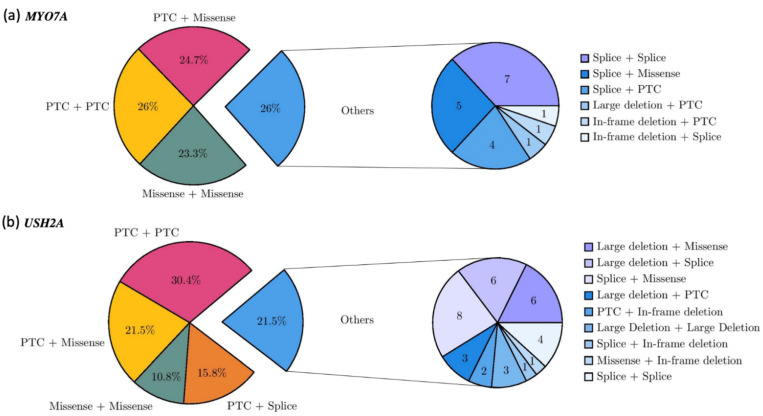
Genotype spectrum of *MYO7A* (**a**) and *USH2A* (**b**). Pie charts on the left showing the frequencies of the different allele combinations. Pie charts on the right showing the number of patients carrying minor combinations of alleles.

In total, 73 patients carried a pathogenic genotype in *MYO7A*. The most frequent type of pathogenic genotype was a combination of two alterations predicting a PTC in 19 patients (26%). Forty patients (54.8%) carried a missense variation in combination with a PTC (18 patients, 24.7%), another missense variation (17 patients, 23.3%) or a splicing alteration (5 patients, 6.8%). Compound splicing alterations were identified in seven patients (9.6%). Four patients carried a splicing allele in combination with a PTC (5.5%) (Figure 4a). 

The *USH2A* pathogenic genotypes were mainly composed of two PTC alleles for 48 of 158 index cases (30.4%). Missense alleles were represented in 41.1% of the total genotypes and were associated with a PTC (n = 34, 21.5%), another missense (n = 17, 10.8%), a splicing alteration (n = 8, 5.1%), a large deletion (n = 6, 3.8%) or an in-frame deletion (n = 1, 0.6%) (Figure 4b). Of note, 48 patients carried at least one of the two major exon 13 pathogenic variants, 41 the c.2299del - p.(Glu767SerfsTer21) variant, and 7 the c.2276G>T - p.(Cys759Phe) variant.

#### 2.2.3. Additional Pathogenic Variant in a Third USH Gene

In the *MYO7A* cohort, five patients carried an additional variant of class 4 or 5, or class 3 with arguments in favor of its pathogenicity lying in a second USH gene. All these variants were heterozygous. In patient U1519, a large duplication involving exons 2 to 12 of the *WHRN* gene was identified by Illumina sequencing and confirmed by aCGH (NM_015404.3:c.(618+1_619−1)_(*674_?)dup, U1519). To date, the extent of the duplication, as well as its location, is unknown. An additional molecular characterization, for example long-read sequencing, would be required to precisely characterize this event. 

In the *USH2A* cohort, 11 patients were found carrying disease-causing or likely disease-causing variants in at least one additional gene. Although some of these rare alleles definitely corresponded to pathogenic alleles (e.g., *USH1G* NM_173477.4:c.800G>A - p.(Trp267Ter)), a number of them consisted of class 3 missense variants (two among the variants reported in other genes linked to the *MYO7A* genotypes, and six in the *USH2A* cohort, Tables S1 and S2).

#### 2.2.4. Clinical Evaluation

During the molecular diagnosis procedure, clinicians are asked to fill in a clinical questionnaire, which lists items including the age of diagnosis and severity of hearing loss, presence of vestibular areflexia and age at diagnosis of RP (including restriction of the visual field, retinitis pigmentosa, fundus examination). Such information is presented in Table S3. The mean age of the *MYO7A* cohort was 32 years versus 43 years for *USH2A* patients. Clinical data for *MYO7A* patients were concordant with type I Usher syndrome. The average age of deafness diagnosis was 4 months (neonatal to 6 years). Gait acquisition was reported in a mean age of 22.5 months, which was concordant with the presence of vestibular areflexia. Night blindness was reported at approximately 9 years of age. Some patients described almost congenital difficulties in the absence of light, but all had visual impairment before their 15th year. RP diagnosis at fundus examination was made at 18.5 years for half the patients. 

Concerning USH2 patients, the age of diagnosis of hearing loss was reported at approximatively 4 years of age (from congenital detection to 37 years). No psychomotor delay was reported for a great majority of patients. However, a 54-year-old male (U1410) was reported with prelingual onset profound hearing loss, in association with vestibular areflexia and motor delay (gait acquisition at 24 months). Somewhat discordantly, RP diagnosis was performed relatively late, at 30 years, but no clinical data was available to estimate the onset of visual impairment. This patient carries though, two typical USH2 variants (c.2299delG and c.7595-2144A>G). Similarly, a 43-year-old female (U1408) with prelingual severe hearing loss was also reported to have gait acquisition delay (24 months), but no evidence of vestibular areflexia was noted during examination. 

## 3. Discussion

In 2015 was published what would become the international standard for DNA variants’ classification, the ACMG/AMP guidelines [13]. These guidelines were meant to be applied to many different genes and conditions, and since then are being continually discussed and adapted [15,16,17,18,19,20].

We report here a set of 24 variants whose classification falls into the unknown significance category (class 3). While there is evidence in favor of their pathogenic effect, such as the combination of PM1, PM2 and PP3 ACMG criteria, it is not sufficient to upgrade to class 4 (likely pathogenic). This shows the need to differentiate in the classification system the variants of truly unknown significance (VUS), from those with evidence in favor of pathogenicity or with particular interest (“hot VUS”) or in favor of a benign effect (“cold VUS”). It is likely that such a differentiation will finally occur even if the precise criteria remain to be established. This raises once again the power of familial studies, as it becomes crucial to provide comprehensive classification of some alterations. Unfortunately, this become complicated when the referred patients are adults, as is the case for a number of cases in this cohort.

The large cohort reported here illustrates once more the known genetic heterogeneity of Usher syndrome due to *MYO7A* and *USH2A* alterations. In addition, this study highlights the implication of missense alleles in Usher syndrome. Moreover, we also show that the combination of two PTCs or two missense alterations can lead to a similar clinical outcome. For example, patients U597, U1241, U1486 and U1809 who carry two *MYO7A* missense alterations present with a typical USH1 phenotype with profound deafness and RP occurring in the second decade of life (Table S3). It is, however, possible that subtle phenotypic variations linked to each variant or combination of variants may be identified upon extensive clinical explorations.

Up to 40% of the *MYO7A* patients carry at least one missense alteration. Consequently, when a patient is newly diagnosed, the chances are that in 40% of cases a missense alteration will be identified, and this study shows that a significant proportion still involve newly described variants. To a lesser extent, but still with a high representation, 28% of *USH2A* patients carry a missense variation. This high proportion of missense alterations reflects the challenges associated with appropriate diagnosis and genetic counselling, as both genes are also implicated in NSHL and isolated RP, respectively.

In this study, we report seven *MYO7A* and five *USH2A* missense alterations that are novel, with in silico predictions compatible with a pathogenic effect, and each alteration identified in *trans* with a pathogenic allele. As this study reports only patients referred with a clinically diagnosed Usher syndrome, these variants can be labelled as USH variants. Nevertheless, thanks to the extent of MPS, large panels that include all USH genes are now offered to analyze young patients referred for NSHL. We previously have shown that in 16% of the cases in our population, pathogenic alterations were identified in an USH gene, most frequently *MYO7A* and *USH2A* [21]. As the contribution of missense variations remains high in USH genotypes for *MYO7A*, and as we cannot predict for either of the two genes whether a new missense alteration will lead to USH or nonsyndromic phenotypes, variant interpretation is more challenging than ever for young patients. It is only by cumulating data from large cohorts together with a rigorous and careful investigation of missense variations to characterize their impact, that an accurate prognosis will be given to the families. New approaches in the field of structural biochemistry, such as normal mode analysis, which has been recently used to assess *WFS1* variants [22], may help discriminate USH variants from NSHL variants.

The prognosis for *USH2A*-linked young patients is quite different, as to date and to our knowledge, patients will develop USH2. The age of onset of the RP though, remains unknown. However, we show here that *USH2A* genotypes with a missense/missense combination also lead to USH in 10% of cases and not to isolated RP, as some studies based on fewer patients would suggest [23]. As expected, the c.2276G>T - p.(Cys759Phe) is present in only 2.2% of our cohort. This variant is known as a hypomorphic allele, and depending of the nature of the second allele, will be linked to USH or isolated RP [24]. Interestingly, in this cohort, in five out of seven patients, the p.(Cys759Phe) allele is present as a complex allele with the c.13274C>T - p.(Thr4425Met) variant. Obviously, the combination of both variants worsens the phenotype, as evidenced by patient U1350 who is homozygous and presents with a typical USH2 syndrome. The p.(Thr4425Met) variant is a recurrent allele present on its own or as a complex allele, therefore, segregation analyses are always necessary to confirm the status of the variants.

The identification of pathogenic genotypes in the two major USH genes is of particular importance, as it would allow the inclusion of eligible patients in ongoing clinical trials. The challenge of treating *MYO7A* and *USH2A* lies in the large size of their cDNAs, which exceeds the cloning capacity of the commonly used vehicles for gene delivery, adenovirus-associated vectors [25]. Nonetheless, a gene supplementation clinical trial (Sanofi, UshStat, NCT01505062) was begun for *MYO7A* in 2012, using an equine infectious anemia lentivirus vector with a larger cloning capacity [26]. This trial has since been terminated, however, and the results have not yet been published. An alternative approach to gene supplementation is currently being developed for *USH2A*. This is based on the use of antisense oligonucleotides (AONs) to induce skipping of exon 13, which harbors the two most prevalent variants, c.2299delG and c.2276G>T (p.Cys759Phe), responsible for USH2 and isolated RP [27]. The AON QR-421a is currently being tested in phase 1/2 trials (ProQR, Stellar, #NCT03780257) for its efficiency in skipping the in-frame exon 13. If successful, nearly a third of our *USH2A* patients could be concerned by this kind of therapy (n = 48, 30.4%). Furthermore, AONs also hold potential as splice-modulation therapies for intronic variants [28], thus opening up the possibility of treating a larger number of *USH2A* mutations, such as the frequent c.7595-2144A>G variant or the novel c.4885+375A>G variant described herein. Therefore, taken together, it is essential that large patient cohorts are fully genotyped, not only to be able to provide the patient with a clear diagnosis, but also in readiness for current and future recruitments into clinical trials.

## 4. Materials and Methods

### 4.1. Patient Recruitment

All patients were referred from Medical Genetics, Ophthalmology or ENT services. All patients underwent a clinical questionnaire for family and were referred for Usher syndrome based on the degree of hearing loss and retinal degeneration. This study was conducted according to the guidelines of the Declaration of Helsinki and in accordance with the French law on bioethics: revised 7 July 2011, number 2011-814. The experimental protocol was approved by the Montpellier University Hospital (CHU Montpellier) as part of the molecular diagnostic activity. The authorization number given by the Agence Régionale de la Santé (ARS) is LR/2013-N°190. Informed consent for genetic testing was obtained from adult probands, or parents in the case of minors, after explanation of the nature and possible implication of the patient and his family. All patients harboring two variants of interest (ACMG class 5, 4, 3 with evidence in favor of pathogenicity) compatible with Usher syndrome were included in this study. When possible, familial segregation was performed. All the presented mutational genotypes are novel.

### 4.2. Molecular Analyses

#### 4.2.1. Gene-Panel Sequencing and Bioinformatics

All probands underwent Ilumina^®^ Massively Parallel Sequencing using a gene-panel approach. The panel included the *MYO7A* (RefSeq accession number NM_000260.3) and *USH2A* (NM_206933.2) genes and was designed using the NimbleGen^®^ SeqCap EZ choice technology. The panel was targeted on exons referenced in RefSeq with 50-bp flanking regions, as well as *USH2A* regions previously described to harbor deep-intronic variants leading to pseudoexon inclusion [14,29]. Sequencing was performed on an Illumina MiniSeq system. The secondary analysis (mainly alignment and variant calling) was performed using the commercial software LocalRunManager (v1.3.1 and v2). Variant Calling Files (VCF) were automatically included in our in-house database system (USHVaM2, https://github.com/beboche/u2), which also performed variant annotation. In addition, when the pathogenic genotype was unclear, the samples were reanalyzed using an in-house pipeline (MobiDL, https://github.com/mobidic/MobiDL), which performs the secondary and tertiary analyses. Copy Number Variations (CNVs) were detected using the MobiCNV algorithm (https://github.com/mobidic/MobiCNV). For one sample (U1432), we performed a complete *USH2A* sequencing, as previously described [29], to screen for deep intronic variants possibly altering proper splicing.

#### 4.2.2. Complementary Analysis

Variants of interest were all confirmed by Sanger sequencing. Potential CNVs were confirmed either by Quantitative Multiplex PCR of Short Fluorescents fragments (QMPSF) or array Comparative Genomic Hybridisation (aCGH). aCGH assays were performed using a Sure Print G3 CGH personal 4 × 180 K custom design for 26 HL genes labelled with the SureTag Complete labelling kit. The array was analyzed on an Agilent DNA microarray scanner C.

### 4.3. Pathogenicity Assessment

The pathogenicity of all variants lying in the *MYO7A* and *USH2A* genes, as well as other variants of interest identified in other genes of the panel (i.e., third pathogenic variants), has been assessed according to the ACMG/AMP guidelines [13]. All reported variants introducing PTCs in this study were considered class 5 (pathogenic, application of the PVS1 criteria according to Tayoun et al., 2018 [30]). Others underwent full assessment, using Intervar [31] as a starter and manual reassessment of the different criteria when necessary.

### 4.4. Functional Analysis

A minigene assay was carried out as previously described [32] in order to validate the in silico splice predictions obtained for the c.4885+375A>G *USH2A* variant. Briefly, PCR-amplified DNA sequences containing the *USH2A* exon 23 flanked by 248 bp of 5′, and 670 bp of 3′ intronic sequences were generated from the proband and a control individual. The pSPL3 exon-trapping vector was used to construct the wild-type and mutant minigenes. Constructions were transfected into HeLa cells using the FuGENE^®^ 6 Transfection Reagent (Promega, Charbonnières-les-Bains, France), according to the manufacturer’s instructions. Twenty-four hours post-transfection, cells were harvested, and RNA was extracted and reverse-transcribed. PCR amplifications were carried out with the SD6 and SA2 pSPL3-specific primers [33]. PCR products were visualized on a 1.5% agarose gel and subsequently Sanger sequenced.

### 4.5. Data Availability

All variants described in this study have been submitted for annotation in MobiDetails [34] and can be retrieved using the following URLS:

*MYO7A*:

https://mobidetails.iurc.montp.inserm.fr/MD/auth/variant_list/MYO7A_LGM_2021 or https://tinyurl.com/yfa9v3h5.

*USH2A*:

https://mobidetails.iurc.montp.inserm.fr/MD/auth/variant_list/USH2A_LGM_2021 or https://tinyurl.com/2y8fjcwu.

In addition, all genotypes have been submitted to the Global Variome Shared LOVD instance (www.lovd.nl/myo7a and www.lovd.nl/ush2a), which gathers variants, genotypes, experiments and phenotypes in all known human genes. Our dataset can be queried using the individual ID ranges #00386958 to #00386974 and #00387143 to #00387357.

## Data Availability

All data are contained within the article or Supplementary Material, or available using the provided URLs.

## Supplementary Materials

The following are available online at https://www.mdpi.com/article/10.3390/ijms222413294/s1.

## Appendix A

UNIPROT domains in Figure A1 and Figure A2 were plotted with IBS, Illustrator for Biological Science (http://ibs.biocuckoo.org) [35].

## Appendix B

**Table A1:** Pathogenic criteria extracted from the ACMG/AMP guidelines. Criteria in bold and italics are the most relevant when studying the *MYO7A* or the *USH2A* genes in an Usher syndrome clinical context (fully penetrant recessive condition). Adapted from [13]. These criteria, taken together with the benign ones, are used to compute a likelihood of pathogenicity, which is translated in a 5-tier terminology system, class 1 being the least pathogenic (benign), and class 5 the most pathogenic.

Evidence of Pathogenicity	Description
**Very strong**	***PVS1 null variant (nonsense, frameshift, canonical ±1 or 2 splice sites, initiation codon, single or multiexon deletion) in a gene where loss of function is a known mechanism of disease***
**Strong**	PS1 Same amino acid change as a previously established pathogenic variant regardless of nucleotide change
	PS2 De novo (both maternity and paternity confirmed) in a patient with the disease and no family history
	***PS3 Well-established*** **in vitro *or* in vivo *functional studies supportive of a damaging effect on the gene or gene product***
	PS4 The prevalence of the variant in affected individuals is significantly increased compared with the prevalence in controls
**Moderate**	***PM1 Located in a mutational hot spot and/or critical and well-established functional domain (e.g., active site of an enzyme) without benign variation***
	***PM2 Absent from controls (or at extremely low frequency if recessive) in Exome Sequencing Project, 1000 Genomes Project, or the Genome Aggregation Database***
	***PM3 For recessive disorders, detected in trans with a pathogenic variant***
	PM4 Protein length changes as a result of in-frame deletions/insertions in a nonrepeat region or stop-loss variants
	***PM5 Novel missense change at an amino acid residue where a different missense change determined to be pathogenic has been seen before***
	PM6 Assumed de novo, but without confirmation of paternity and maternity
**Supporting**	***PP1 Cosegregation with disease in multiple affected family members in a gene definitively known to cause the disease***
	PP2 Missense variant in a gene that has a low rate of benign missense variation and in which missense variants are a common mechanism of disease
	***PP3 Multiple lines of computational evidence support a deleterious effect on the gene or gene product (conservation, evolutionary, splicing impact, etc.)***
	PP4 Patient’s phenotype or family history is highly specific for a disease with a single genetic etiology
	***PP5 Reputable source recently reports variant as pathogenic, but the evidence is not available to the laboratory to perform an independent evaluation***

## References

[1] Millán J.M., Aller E., Jaijo T., Blanco-Kelly F., Gimenez-Pardo A., Ayuso C. (2011). An Update on the Genetics of Usher Syndrome. J. Ophthalmol..

[2] Le Quesne Stabej P., Saihan Z., Rangesh N., Steele-Stallard H.B., Ambrose J., Coffey A., Emmerson J., Haralambous E., Hughes Y., Steel K.P. (2012). Comprehensive sequence analysis of nine Usher syndrome genes in the UK National Collaborative Usher Study. J. Med. Genet..

[3] Roux A.-F., Faugère V., Vaché C., Baux D., Besnard T., Léonard S., Blanchet C., Hamel C., Mondain M., Gilbert-Dussardier B. (2011). Four-Year Follow-up of Diagnostic Service in USH1 Patients. Investig. Opthalmol. Vis. Sci..

[4] Bonnet C., Riahi Z., Chantot-Bastaraud S., Smagghe L., Letexier M., Marcaillou C., Lefèvre G.M., Hardelin J.-P., El-Amraoui A., Singh-Estivalet A. (2016). An innovative strategy for the molecular diagnosis of Usher syndrome identifies causal biallelic mutations in 93% of European patients. Eur. J. Hum. Genet..

[5] Abadie C., Blanchet C., Baux D., Larrieu L., Besnard T., Ravel P., Biboulet R., Hamel C., Malcolm S., Mondain M. (2011). Audiological findings in 100 USH2 patients. Clin. Genet..

[6] Besnard T., Vaché C., Baux D., Larrieu L., Abadie C., Blanchet C., Odent S., Blanchet P., Calvas P., Hamel C. (2012). Non-USH2A mutations in USH2 patients. Hum. Mutat..

[7] García-García G., Besnard T., Baux D., Vaché C., Aller E., Malcolm S., Claustres M., Millan J.M., Roux A.-F. (2013). The contribution of GPR98 and DFNB31 genes to a Spanish Usher syndrome type 2 cohort. Mol. Vis..

[8] Shearer A.E., Hildebrand M.S., Smith R.J., Adam M.P., Ardinger H.H., Pagon R.A., Wallace S.E., Bean L.J., Mirzaa G., Amemiya A. (1993). Hereditary Hearing Loss and Deafness Overview. GeneReviews^®^.

[9] Kabahuma R., Schubert W.-D., Labuschagne C., Yan D., Blanton S., Pepper M., Liu X. (2021). Spectrum of *MYO7A* Mutations in an Indigenous South African Population Further Elucidates the Nonsyndromic Autosomal Recessive Phenotype of DFNB2 to Include Both Homozygous and Compound Heterozygous Mutations. Genes.

[10] Colombo L., Maltese P.E., Castori M., El Shamieh S., Zeitz C., Audo I., Zulian A., Marinelli C., Benedetti S., Costantini A. (2021). Molecular Epidemiology in 591 Italian Probands With Nonsyndromic Retinitis Pigmentosa and Usher Syndrome. Investig. Opthalmol. Vis. Sci..

[11] Fuster-García C., García-García G., Jaijo T., Fornés N., Ayuso C., Fernández-Burriel M., La Morena A.S.-D., Aller E., Millán J.M. (2018). High-throughput sequencing for the molecular diagnosis of Usher syndrome reveals 42 novel mutations and consolidates CEP250 as Usher-like disease causative. Sci. Rep..

[12] Neuhaus C., Eisenberger T., Decker C., Nagl S., Blank C., Pfister M., Kennerknecht I., Müller-Hofstede C., Issa P.C., Heller R. (2017). Next-generation sequencing reveals the mutational landscape of clinically diagnosed Usher syndrome: Copy number variations, phenocopies, a predominant target for translational read-through, andPEX26mutated in Heimler syndrome. Mol. Genet. Genom. Med..

[13] Richards S., Aziz N., Bale S., Bick D., Das S., Gastier-Foster J., Grody W.W., Hegde M., Lyon E., Spector E. (2015). Standards and guidelines for the interpretation of sequence variants: A joint consensus recommendation of the American College of Medical Genetics and Genomics and the Association for Molecular Pathology. Genet. Med..

[14] Vaché C., Besnard T., le Berre P., García-García G., Baux D., Larrieu L., Abadie C., Blanchet C., Bolz H.J., Millan J. (2011). Usher syndrome type 2 caused by activation of an USH2A pseudoexon: Implications for diagnosis and therapy. Hum. Mutat..

[15] Patel M.J., DiStefano M.T., Oza A.M., Hughes M.Y., Wilcox E.H., Hemphill S.E., Cushman B.J., Grant A.R., Siegert R.K., Shen J. (2021). Disease-specific ACMG/AMP guidelines improve sequence variant interpretation for hearing loss. Genet. Med..

[16] van der Sluijs P.J., Alders M., Dingemans A.J.M., Parbhoo K., van Bon B.W., Dempsey J.C., Doherty D., Dunnen J.T.D., Gerkes E.H., Milller I.M. (2021). A Case Series of Familial *ARID1B* Variants Illustrating Variable Expression and Suggestions to Update the ACMG Criteria. Genes.

[17] Oza A.M., DiStefano M.T., Hemphill S.E., Cushman B.J., Grant A.R., Siegert R.K., Shen J., Chapin A., Boczek N.J., Schimmenti L.A. (2018). Expert specification of the ACMG/AMP variant interpretation guidelines for genetic hearing loss. Hum. Mutat..

[18] Kountouris P., Stephanou C., Lederer C.W., Traeger-Synodinos J., Bento C., Harteveld C.L., Fylaktou E., Koopmann T.T., Halim-Fikri H., Michailidou K. (2021). Adapting the ACMG/AMP variant classification framework: A perspective from the ClinGen Hemoglobinopathy Variant Curation Expert Panel. Hum. Mutat..

[19] Davieson C.D., Joyce K.E., Sharma L., Shovlin C.L. (2021). DNA variant classification–reconsidering “allele rarity” and “phenotype” criteria in ACMG/AMP guidelines. Eur. J. Med Genet..

[20] Brandt T., Sack L.M., Arjona D., Tan D., Mei H., Cui H., Gao H., Bean L.J.H., Ankala A., Del Gaudio D. (2019). Adapting ACMG/AMP sequence variant classification guidelines for single-gene copy number variants. Genet. Med..

[21] Baux D., Vaché C., Blanchet C., Willems M., Baudoin C., Moclyn M., Faugère V., Touraine R., Isidor B., Dupin-Deguine D. (2017). Combined genetic approaches yield a 48% diagnostic rate in a large cohort of French hearing-impaired patients. Sci. Rep..

[22] Wilf-Yarkoni A., Shor O., Fellner A., Hellmann M.A., Pras E., Yonath H., Shkedi-Rafid S., Basel-Salmon L., Bazak L., Eliahou R. (2021). Mild Phenotype of Wolfram Syndrome Associated With a Common Pathogenic Variant Is Predicted by a Structural Model of Wolframin. Neurol. Genet..

[23] Reurink J., Dockery A., Oziębło D., Farrar G., Ołdak M., Brink J.T., Bergen A., Rinne T., Yntema H., Pennings R. (2021). Molecular Inversion Probe-Based Sequencing of *USH2A* Exons and Splice Sites as a Cost-Effective Screening Tool in USH2 and arRP Cases. Int. J. Mol. Sci..

[24] Pérez-Carro R., Blanco-Kelly F., Galbis-Martínez L., García-García G., Aller E., García-Sandoval B., Mínguez P., Corton M., Mahíllo-Fernández I., Martín-Mérida I. (2018). Unravelling the pathogenic role and genotype-phenotype correlation of the USH2A p.(Cys759Phe) variant among Spanish families. PLoS ONE.

[25] Trapani I., Auricchio A. (2019). Has retinal gene therapy come of age? From bench to bedside and back to bench. Hum. Mol. Genet..

[26] Zallocchi M., Binley K., Lad Y., Ellis S., Widdowson P., Iqball S., Scripps V., Kelleher M., Loader J., Miskin J. (2014). EIAV-Based Retinal Gene Therapy in the shaker1 Mouse Model for Usher Syndrome Type 1B: Development of UshStat. PLoS ONE.

[27] Dulla K., Slijkerman R., van Diepen H.C., Albert S., Dona M., Beumer W., Turunen J.J., Chan H.L., Schulkens I.A., Vorthoren L. (2021). Antisense oligonucleotide-based treatment of retinitis pigmentosa caused by USH2A exon 13 mutations. Mol. Ther..

[28] Gerard X., Garanto A., Rozet J.-M., Collin R.W.J. (2016). Antisense Oligonucleotide Therapy for Inherited Retinal Dystrophies. Adv. Exp. Med. Biol..

[29] Liquori A., Vaché C., Baux D., Blanchet C., Hamel C., Malcolm S., Koenig M., Claustres M., Roux A.-F. (2015). WholeUSH2AGene Sequencing Identifies Several New Deep Intronic Mutations. Hum. Mutat..

[30] Abou Tayoun A.N., Pesaran T., DiStefano M.T., Oza A., Rehm H.L., Biesecker L.G., Harrison S.M. (2018). ClinGen Sequence Variant Interpretation Working Group (ClinGen SVI) Recommendations for interpreting the loss of function PVS1 ACMG/AMP variant criterion. Hum. Mutat..

[31] Li Q., Wang K. (2017). InterVar: Clinical Interpretation of Genetic Variants by the 2015 ACMG-AMP Guidelines. Am. J. Hum. Genet..

[32] Le Guédard-Méreuze S., Vaché C., Baux D., Faugère V., Larrieu L., Abadie C., Janecke A., Claustres M., Roux A.-F., Tuffery-Giraud S. (2010). Ex vivo splicing assays of mutations at noncanonical positions of splice sites in USHER genes. Hum. Mutat..

[33] Bottillo I., De Luca A., Schirinzi A., Guida V., Torrente I., Calvieri S., Gervasini C., Larizza L., Pizzuti A., Dallapiccola B. (2007). Functional analysis of splicing mutations in exon 7 of NF1gene. BMC Med. Genet..

[34] Baux D., Van Goethem C., Ardouin O., Guignard T., Bergougnoux A., Koenig M., Roux A.-F. (2020). MobiDetails: Online DNA variants interpretation. Eur. J. Hum. Genet..

[35] Wenzhong L., Yubon X., Jiyong M., Xiaotong L., Peng N., Zhixiang Z., Lahrmann U., Zhao Q., Zheng Y., Zhao Y. (2015). IBS: An illustrator for the presentation and visualization of biological sequences. Bioinformatics.

